# Supplementary material for: A nuclear-based quality control pathway for non-imported mitochondrial proteins
Source: eLife. 2021 Mar 18;10:e61230. doi: 10.7554/eLife.61230 (PMC7993989; doi:10.7554/eLife.61230)
Supplement: Supplementary file 2. [file elife-61230-supp2.docx]

| Strain | Genotype |
| --- | --- |
| BY4741 | MATa his3∆ leu2∆ ura3∆ met15∆ |
| AHY3354 | MATa his3∆ leu2∆ ura3∆ met15∆ TOM70-mCherry:KanMX MIR1-yeGFP:HisMX |
| AHY3742 | MATa his3∆ leu2∆ ura3∆ met15∆ TOM70-mCherry:KanMX COX15-yeGFP:HisMX |
| AHY3746 | MATa his3∆ leu2∆ ura3∆ met15∆ TOM70-mCherry:KanMX Idh1-yeGFP:HisMX |
| AHY3746 | MATa his3∆ leu2∆ ura3∆ met15∆ TOM70-mCherry:KanMX LAT1-yeGFP:HisMX |
| AHY3857 | MATa his3∆ leu2∆ ura3∆ met15∆ TOM70-mCherry:KanMX COX15-yeGFP:HisMX pdr5Δ::URA3 |
| AHY3861 | MATa his3∆ leu2∆ ura3∆ met15∆ TOM70-mCherry:KanMX LAT1-yeGFP:HisMX pdr5Δ::URA3 |
| AHY3934 | MATa/MATa his3∆1/his3∆1 leu2∆0/leu2∆0 ura3∆0/ura3∆0 lys2∆0/+ met15∆0/+ Term_cyc1_:URA3-P_GPD/TDH3_-cre-EBD78:Term_cyc1_/+ ILV2-V5-loxP-HA-GFP-HygX-loxP-T7-mRFP-KanMX/+ |
| AHY4042 | MATa his3∆ leu2∆ ura3∆ met15∆ ILV2-yeGFP:HisMX |
| AHY4389 | MATa his3∆ leu2∆ ura3∆ met15∆ ILV2-yeGFP:HisMX pdr5Δ::URA3 |
| AHY4628 | MATa his3∆ leu2∆ met15∆ URA3::CMV-tTA TOM70-mCherry:KanMX ILV2-yeGFP:HisMX |
| AHY4737 | MATa his3∆ leu2∆ ura3∆ met15∆ TOM70-mCherry:KanMX ACP1-yeGFP:HisMX |
| AHY4739 | MATa his3∆ leu2∆ ura3∆ met15∆ ILV2-yeGFP:HisMX TOM70-mCherry:KanMX |
| AHY4945 | MATa his3∆ leu2∆ ura3∆ met15∆ ILV2-3xHA:HisMX |
| AHY4949 | MATa his3∆ leu2∆ ura3∆ met15∆ DLD1-yeGFP:HisMX |
| AHY4951 | MATa his3∆ leu2∆ ura3∆ met15∆ DLD2-yeGFP:HisMX |
| AHY4959 | MATa his3∆ leu2∆ ura3∆ met15∆ DLD1-yeGFP:HisMX pdr5Δ::URA3 |
| AHY4961 | MATa his3∆ leu2∆ ura3∆ met15∆ DLD2-yeGFP:HisMX pdr5Δ::URA3 |
| AHY4963 | MATa his3∆ leu2∆ met15∆ URA3::CMV-tTA TOM70-mCherry:KanMX DLD1-yeGFP:HisMX |
| AHY4965 | MATa his3∆ leu2∆ met15∆ URA3::CMV-tTA TOM70-mCherry:KanMX DLD2-yeGFP:HisMX |
| AHY4971 | MATa his3∆ leu2∆ ura3∆ met15∆ ILV2-3xHA:HisMX pdr5Δ::URA3 |
| AHY5044 | MATa his3∆ leu2∆ ura3∆ met15∆ san1Δ::NatMX |
| AHY5047 | MATa his3∆ leu2∆ ura3∆ lys2Δ ubr1Δ::URA3 doa10Δ::HygMX |
| AHY5048 | MATa his3∆ leu2∆ ura3∆ met15∆ doa10Δ::HygMX |
| AHY5049 | MATa his3∆ leu2∆ ura3∆ met15∆ lys2Δ san1Δ::NatMX doa10Δ::HygMX |
| AHY5053 | MATa his3∆ leu2∆ ura3∆ lys2∆ ubr1Δ::URA3 |
| AHY5055 | MATa his3∆ leu2∆ ura3∆ met15∆ san1Δ::NatMX ubr1Δ::URA3 |
| AHY5056 | MATa his3∆ leu2∆ ura3∆ met15∆ lys2Δ san1Δ::NatMX ubr1Δ::URA3 doa10Δ::HygMX |
| AHY5058 | MATa his3∆ leu2∆ ura3∆ met15∆ lys2Δ san1Δ::NatMX ubr1Δ::URA3 doa10Δ::HygMX DLD1-yeGFP:HisMX |
| AHY5060 | MATa his3∆ leu2∆ ura3∆ met15∆ lys2Δ san1Δ::NatMX ubr1Δ::URA3 doa10Δ::HygMX DLD2-yeGFP:HisMX |
| AHY5062 | MATa his3∆ leu2∆ ura3∆ met15∆ lys2Δ san1Δ::NatMX ubr1Δ::URA3 doa10Δ::HygMX ILV2-yeGFP:HisMX |
| AHY6027 | MATa his3∆ leu2∆ ura3∆ met15∆ san1Δ::NatMX DLD1-yeGFP:HisMX |
| AHY6029 | MATa his3∆ leu2∆ ura3∆ lys2∆ ubr1Δ::URA3 DLD1-yeGFP:HisMX |
| AHY6031 | MATa his3∆ leu2∆ ura3∆ met15∆ doa10Δ::HygMX DLD1-yeGFP:HisMX |
| AHY6033 | MATa his3∆ leu2∆ ura3∆ met15∆ san1Δ::NatMX ubr1Δ::URA3 DLD1-GFP:HisMX |
| AHY6035 | MATa his3∆ leu2∆ ura3∆ met15∆ lys2Δ san1Δ::NatMX doa10Δ::HygMX DLD1-yeGFP:HisMX |
| AHY6037 | MATa his3∆ leu2∆ ura3∆ lys2Δ ubr1Δ::URA3 doa10Δ::HygMX DLD1-yeGFP:HisMX |
| AHY6039 | MATa his3∆ leu2∆ ura3∆ met15∆ san1Δ::NatMX DLD2-yeGFP:HisMX |
| AHY6041 | MATa his3∆ leu2∆ ura3∆ lys2∆ ubr1Δ::URA3 DLD2-yeGFP:HisMX |
| AHY6043 | MATa his3∆ leu2∆ ura3∆ met15∆ doa10Δ::HygMX DLD2-yeGFP:HisMX |
| AHY6045 | MATa his3∆ leu2∆ ura3∆ met15∆ san1Δ::NatMX ubr1Δ::URA3 DLD2-yeGFP:HisMX |
| AHY6047 | MATa his3∆ leu2∆ ura3∆ met15∆ lys2Δ san1Δ::NatMX doa10Δ::HygMX DLD2-yeGFP:HisMX |
| AHY6049 | MATa his3∆ leu2∆ ura3∆ lys2Δ ubr1Δ::URA3 doa10Δ::HygMX DLD2-yeGFP:HisMX |
| AHY6051 | MATa his3∆ leu2∆ ura3∆ met15∆ san1Δ::NatMX ILV2-yeGFP:HisMX |
| AHY6053 | MATa his3∆ leu2∆ ura3∆ lys2∆ ubr1Δ::URA3 ILV2-yeGFP:HisMX |
| AHY6055 | MATa his3∆ leu2∆ ura3∆ met15∆ doa10Δ::HygMX ILV2-yeGFP:HisMX |
| AHY6057 | MATa his3∆ leu2∆ ura3∆ met15∆ san1Δ::NatMX ubr1Δ::URA3 ILV2-yeGFP:HisMX |
| AHY6059 | MATa his3∆ leu2∆ ura3∆ met15∆ lys2Δ san1Δ::NatMX doa10Δ::HygMX ILV2-yeGFP:HisMX |
| AHY6061 | MATa his3∆ leu2∆ ura3∆ lys2Δ ubr1Δ::URA3 doa10Δ::HygMX ILV2-yeGFP:HisMX |
| AHY6063 | MATa his3∆ leu2∆ ura3∆ met15∆ lys2Δ san1Δ::NatMX ubr1Δ::URA3 doa10Δ::HygMX LAT1-yeGFP:HisMX |
| AHY6408 | MATa his3∆ leu2∆ ura3∆ met15∆ lys2Δ san1Δ::NatMX ubr1Δ::URA3 doa10Δ::HygMX ΔPDR5::G418, ILV2-3xHA:HixMX |
| AHY6802 | MATa his3∆ leu2∆ met15∆ P_TOM40_::NatMX-tet07-TATA URA3::CMV-tTA TOM70-mCherry:KanMX ILV2-yeGFP:HisMX |
| AHY6804 | MATa his3∆ leu2∆ met15∆ P_TOM40_::NatMX-tet07-TATA URA3::CMV-tTA TOM70-mCherry:KanMX DLD1-yeGFP:HisMX |
| AHY6806 | MATa his3∆ leu2∆ met15∆ P_TOM40_::NatMX-tet07-TATA URA3::CMV-tTA TOM70-mCherry:KanMX DLD2-GFP:HisMX |
| AHY6808 | MATa his3∆ leu2∆ met15∆ P_TOM40_::NatMX-tet07-TATA URA3::CMV-tTA TOM70-mCherry:KanMX MIR1-yeGFP:HisMX |
| AHY6864 | MATa his3∆ leu2∆ met15∆ P_TOM40_::NatMX-tet07-TATA URA3::CMV-tTA TOM70-mCherry:KanMX TOM20-yeGFP:HisMX |
| AHY6867 | MATa his3∆ leu2∆ met15∆ P_TOM40_::NatMX-tet07-TATA URA3::CMV-tTA TOM70-mCherry:KanMX COX15-yeGFP:HisMX |
| AHY6870 | MATa his3∆ leu2∆ met15∆ P_TOM40_::NatMX-tet07-TATA URA3::CMV-tTA TOM70-mCherry:KanMX ACP1-yeGFP:HisMX |
| AHY6948 | MATa his3∆ leu2∆ ura3∆ met15∆ SEC61-mCherry:KanMX MIR1-yeGFP:HisMX |
| AHY7181 | MATa his3∆ leu2∆ met15∆ URA3::CMV-tTA TOM70-mCherry:KanMX ACP1-yeGFP:HisMX |
| AHY7183 | MATa his3∆ leu2∆ met15∆ URA3::CMV-tTA TOM70-mCherry:KanMX MIR1-yeGFP:HisMX |
| AHY7187 | MATa his3∆ leu2∆ met15∆ URA3::CMV-tTA TOM70-mCherry:KanMX COX15-yeGFP:HisMX |
| AHY7226 | MATa his3∆ leu2∆ ura3∆ met15∆ TOM70-mCherry:KanMX pRS413-pGPD-COX15-GFP |
| AHY7228 | MATa his3∆ leu2∆ ura3∆ met15∆ TOM70-mCherry:KanMX pRS413-GPD-∆MTS (∆N1-65) COX15-GFP |
| AHY7582 | MATa his3∆ leu2∆ ura3∆ met15∆ TOM70-mCherry:KanMX TOM20-yeGFP:HisMX |
| AHY7584 | MATa his3∆ leu2∆ ura3∆ met15∆ TOM70-mCherry:KanMX DLD1-yeGFP:HisMX |
| AHY7586 | MATa his3∆ leu2∆ ura3∆ met15∆ TOM70-mCherry:KanMX DLD2-yeGFP:HisMX |
| AHY7594 | MATa his3∆ leu2∆ ura3∆ met15∆ lys2Δ san1Δ::NatMX ubr1Δ::URA3 doa10Δ::HygMX DLD1-yeGFP:HisMX TOM70-mCherry:KanMX |
| AHY7596 | MATa his3∆ leu2∆ ura3∆ met15∆ lys2Δ san1Δ::NatMX ubr1Δ::URA3 doa10Δ::HygMX DLD2-yeGFP:HisMX TOM70-mCherry:KanMX |
| AHY7598 | MATa his3∆ leu2∆ ura3∆ met15∆ lys2Δ san1Δ::NatMX, ubr1Δ::URA3 doa10Δ::HygMX ILV2-yeGFP:HisMX TOM70-mCherry:KanMX |
| AHY7742 | MATa his3∆ leu2∆ met15∆ URA3::CMV-tTA TOM70-mCherry:KanMX TOM20-yeGFP:HisMX |
| AHY7875 | MATa his3∆ leu2∆ ura3∆ met15∆ TOM70-mCherry:KanMX pRS413-GPD-∆MTS (∆N1-55)ILV2-GFP |
| AHY7876 | MATa his3∆ leu2∆ ura3∆ met15∆ TOM70-mCherry:KanMX pRS413-GPD-MTS_ILV2_-GFP |
| AHY7965 | MATa his3∆ leu2∆ ura3∆ met15∆ TOM70-mCherry:KanMX pRS413-pGPD-MTS_COX15_-GFP |
| AHY7967 | MATa his3∆ leu2∆ ura3∆ met15∆ TOM70-mCherry:KanMX pRS413-GPD-∆MTS (∆N 1-28) LAT1-GFP |
| AHY7969 | MATa his3∆ leu2∆ ura3∆ met15∆ TOM70-mCherry:KanMX pRS413-pGPD-LAT1-GFP |
| AHY8001 | MATa his3∆ leu2∆ ura3∆ met15∆ lys2Δ san1Δ::NatMX, ubr1Δ::URA3 doa10Δ::HygMX TOM70-mCherry:KanMX pRS413-GPD-∆MTS (∆N1-55)ILV2-GFP |
| AHY8003 | MATa his3∆ leu2∆ ura3∆ met15∆ lys2Δ san1Δ::NatMX ubr1Δ::URA3 doa10Δ::HygMX TOM70-mCherry:KanMX pRS413-pGPD-MTS_ILV2_-GFP |
| AHY8008 | MATa his3∆ leu2∆ ura3∆ met15∆ TOM70-mCherry:KanMX pRS413-pGPD-MTS_LAT1_-GFP |
| AHY8027 | MATa his3∆ leu2∆ ura3∆ met15∆ TOM70-mCherry:KanMX pRS413-pGPD-ILV2-GFP |
| AHY8031 | MATa his3∆ leu2∆ ura3∆ met15∆ lys2Δ san1Δ::NatMX ubr1Δ::URA3 doa10Δ::HygMX TOM70-mCherry:KanMX pRS413-pGPD-ILV2-GFP |
| AHY8043 | MATa his3∆ leu2∆ ura3∆ met15∆ lys2Δ san1Δ::NatMX ubr1Δ::URA3 doa10Δ::HygMX Tom70-mCherry:KanMX pRS413-pGPD-DLD2-GFP |
| AHY8345 | MATa his3∆ leu2∆ ura3∆ met15∆ TOM70-GFP:KanMX TIM50-mCherry:KanMX ilv2∆::URA3 |
| AHY8557 | MATa his3∆ leu2∆ ura3∆ met15∆ TOM70-mCherry:KanMX pRS413-GPD-∆MTS (∆N1-35) DLD2-GFP |
| AHY8559 | MATa his3∆ leu2∆ ura3∆ met15∆ lys2Δ san1Δ::NatMX, ubr1Δ::URA3 doa10Δ::HygMX TOM70-mCherry:KanMX pRS413-GPD-∆MTS (∆N1-35) DLD2-GFP |
| AHY8561 | MATa his3∆ leu2∆ ura3∆ met15∆ TOM70-mCherry-KanMX pRS413-pGPD-MTS_DLD2_-GFP |
| AHY8563 | MATa his3∆ leu2∆ ura3∆ met15∆ lys2Δ san1Δ::NatMX ubr1Δ::URA3 doa10Δ::HygMX TOM70-mCherry:KanMX pRS413-pGPD-MTS_DLD2_-GFP |
| AHY8671 | MATa his3∆ leu2∆ ura3∆ met15∆ TOM70-mCherry:KanMX pRS413-pGPD-DLD2-GFP |
| AHY10107 | MATa his3∆ leu2∆ ura3∆ met15∆ lys2ΔTOM70-mCherry KanMX pdr5Δ::URA3 LAT1-3XHA-HisMX |
| AHY10198 | MATa his3∆ leu2∆ ura3∆ met15∆ ILV2-3xHA:KanMX pdr5Δ::Ura3 NUP49-yeGFP:HisMX |
| AHY10267 | MATa his3∆ leu2∆ ura3∆ met15∆ ACP1-3xHA:HisMX |
| AHY10269 | MATa his3∆ leu2∆ ura3∆ met15∆ COX15-3xHA:HisMX |
| AHY10369 | MATa his3∆ leu2∆ ura3∆ met15∆ ILV2-5FLAG:KanMX TOM70-mCherry:HygMX |
| AHY10371 | MATa his3∆ leu2∆ ura3∆ met15∆ TOM20-5FLAG:KanMX TOM70-mCherry:HygMX |
| AHY10373 | MATa his3∆ leu2∆ ura3∆ met15∆ MIR1-5FLAG:KanMX TOM70-mCherry:HygMX |
| AHY10375 | MATa his3∆ leu2∆ ura3∆ met15∆ ACP1-5FLAG:KanMX TOM70-mCherry:HygMX |
| AHY10377 | MATa his3∆ leu2∆ ura3∆ met15∆ COX15-5FLAG:KanMX TOM70-mCherry:HygMX |
| AHY10381 | MATa his3∆ leu2∆ ura3∆ met15∆ MIR1-3xHA:HisMX |
| AHY10385 | MATa his3∆ leu2∆ ura3∆ met15∆ TOM20-3xHA:HisMX TOM70-mCherry:KanMX |
| AHY10437 | MATa his3∆ leu2∆ ura3∆ met15∆ COX15-3xHA:HisMX pdr5Δ::URA3 |
| AHY10891 | MATa his3∆ leu2∆ ura3∆ met15∆ TOM70-mCherry:KanMX ILV2-NES-yeGFP:HisMX |
| AHY10893 | MATa his3∆ leu2∆ ura3∆ met15∆ TOM70-mCherry:KanMX pRS413-pGPD-ILV2 |
| AHY10956 | MATa his3∆ leu2∆ ura3∆ met15∆ TOM70-mCherry:KanMX Idh1-yeGFP:NatMX pRS413-pGPD-Ilv2 |
| AHY10998 | MATa his3∆ leu2∆ ura3∆ met15∆ TOM70-mCherry:KanMX pRS413-GPD-∆MTS (∆N1-55) ILV2-GFP |
| AHY11000 | MATa his3∆ leu2∆ ura3∆ met15∆ TOM70-mCherry:KanMX pRS413-GPD-MTS_ILV2_ |
