# Supplementary material for: A nuclear-based quality control pathway for non-imported mitochondrial proteins
Source: eLife. 2021 Mar 18;10:e61230. doi: 10.7554/eLife.61230 (PMC7993989; doi:10.7554/eLife.61230)
Supplement: Supplementary file 3. [file elife-61230-supp3.docx]

| **Name** | **Number** | **Sequence** |
| --- | --- | --- |
| **Tagging Primers** | | |
| NUP49 pKT F5 | 2260 | GTTACATCAAAAAACGAAAACACTGGCATCATTGAGCATAGGTGACGGTGCTGGTTTA |
| NUP49 pKT R3 | 2261 | ACTTGTTATACGCACTATATAAACTTTCAGGGCGATTTACTCGATGAATTCGAGCTCG |
| TOM20 pKT F5 | 465 | GCCGAATCTGATGCGGTTGCTGAAGCTAACGATATCGATGACGGTGACGGTGCTGGTTTA |
| TOM20 pKT R3 | 466 | AAGAAACAAAAACGGAGAAAAAAAGCAAGCAAAATGTTACTCTCGATGAATTCGAGCTCG |
| ILV2 pKT F5 | 1481 | ACAGACTGAATTACGTCATAAGCGTACAGGCGGTAAGCACGGTGACGGTGCTGGTTTA |
| ILV2 pKT R3 | 1482 | TTTTTACTGAAAATGCTTTTGAAATAAATGTTTTTGAAATTCGATGAATTCGAGCTCG |
| MIR1 pKT F5 | 1556 | GGGTTGCCCACCAACCATTGAAATTGGTGGTGGTGGTCATGGTGACGGTGCTGGTTTA |
| MIR1 pKT R3 | 1557 | GAGGAGAGAATATATATGCATGTATCAATCAAGACCATTTTCGATGAATTCGAGCTCG |
| LAT1 pKT F5 | 1806 | ATTGAAAACTGTTATTGAAAATCCTTTGGAAATGCTATTGGGTGACGGTGCTGGTTTA |
| LAT1 pKT R3 | 1807 | AGATACGCATTTACTGGCGAATTTTATTTTCATTCTAACCTCGATGAATTCGAGCTCG |
| COX15 pKT F5 | 1809 | AATTTTAAGTGAAGCGTCGAAGTTAGCCTCGAAACCATTAGGTGACGGTGCTGGTTTA |
| COX15 pKT R3 | 1810 | GCGAGTATACTGTCAATTCTCATAAGAATACCTTTATCCATCGATGAATTCGAGCTCG |
| ILV2 RITE F5 | 1831 | ACAGACTGAATTACGTCATAAGCGTACAGGCGGTAAGCACGGTGGATCTGGTGGATCT |
| ILV2 RITE R3 | 1832 | TTTTTACTGAAAATGCTTTTGAAATAAATGTTTTTGAAATTTAGGCGCCGGTGGAGTGGCG |
| DLD1 pKT F5 | 2271 | CTTTAAAACTGATCCAAACGAGCCCGCTAATGATTACAGGGGTGACGGTGCTGGTTTA |
| DLD1 pKT R3 | 2272 | TTCAGGTTTACGTGAAGGGTGAAAAAGGAAAATCAGATACTCGATGAATTCGAGCTCG |
| DLD2 pKT F5 | 2274 | TTATGATCCTAATGGAATTTTAAACCCTTACAAATACATTGGTGACGGTGCTGGTTTA |
| DLD2 pKT R3 | 2275 | TATACATATGTAGATAACTATAAAACTTGGCATTTTATTTTCGATGAATTCGAGCTCG |
| SEC61 pKT F5 | 2836 | GTTTACTAAGAACCTCGTTCCAGGATTTTCTGATTTGATGGGTGACGGTGCTGGTTTA |
| SEC61 pKT R3 | 2837 | GCGATTTTTTTTTTCTTTGGATATTATTTTCATTTTATATTCGATGAATTCGAGCTCG |
| ACP1 pKT F5 | 2169 | TGAAACGGTCGATTATATCGCTTCCAATCCCGACGCAAACGGTGACGGTGCTGGTTTA |
| ACP1 pKT R3 | 2170 | GGGGTGACACGATACAATATAATAGAGCGGGGACGGACACTCGATGAATTCGAGCTCG |
| TOM20 pFA6 F5 | 3959 | CGAATCTGATGCGGTTGCTGAAGCTAACGATATCGATGACCGGATCCCCGGGTTAATTAA |
| TOM20 pFA6 F5 | 3960 | GAAACAAAAACGGAGAAAAAAAGCAAGCAAAATGTTACTCGAATTCGAGCTCGTTTAAAC |
| TOM20 chk | 3961 | CAGCTCTATCAGCCACCGGTTATGCTATCT |
| ACP1 pFA6 F5 | 3955 | TGAAACGGTCGATTATATCGCTTCCAATCCCGACGCAAACCGGATCCCCGGGTTAATTAA |
| ACP1 pFA6 F5 | 3956 | GGGGTGACACGATACAATATAATAGAGCGGGGACGGACACGAATTCGAGCTCGTTTAAAC |
| ACP1 chk | 2171 | CAACACAACTAACTCAATACAGCACCTTCC |
| MIR1 pFA6 F5 | 4079 | GGGTTGCCCACCAACCATTGAAATTGGTGGTGGTGGTCATCGGATCCCCGGGTTAATTAA |
| MIR1 pFA6 R3 | 4080 | GAGGAGAGAATATATATGCATGTATCAATCAAGACCATTTGAATTCGAGCTCGTTTAAAC |
| Mir1 chk | 1558 | AGCAGACACTCTGTTGTCCAAGGTCAACAA |
| ILV2 pFA6 F5 | 2210 | ACAGACTGAATTACGTCATAAGCGTACAGGCGGTAAGCACCGGATCCCCGGGTTAATTAA |
| ILV2 pFA6 R3 | 2211 | TTTTTACTGAAAATGCTTTTGAAATAAATGTTTTTGAAATGAATTCGAGCTCGTTTAAAC |
| ILV2 chk | 561 | TTGGTTATTGACATTGATGGTGACGCATCC |
| COX15 pFA6 F5 | 3957 | AATTTTAAGTGAAGCGTCGAAGTTAGCCTCGAAACCATTACGGATCCCCGGGTTAATTAA |
| COX15 pFA6 R3 | 3958 | GCGAGTATACTGTCAATTCTCATAAGAATACCTTTATCCAGAATTCGAGCTCGTTTAAAC |
| COX15 chk | 1811 | AATGGGTGAACGATGGTTCCCTAGTTCTCG |
| LAT1 pFA6 F5 | 2885 | ATTGAAAACTGTTATTGAAAATCCTTTGGAAATGCTATTGCGGATCCCCGGGTTAATTAA |
|  |  |  |
| LAT1 pFA6 R3 | 2886 | AGATACGCATTTACTGGCGAATTTTATTTTCATTCTAACCGAATTCGAGCTCGTTTAAAC |
| LAT1 chk | 1808 | GCCAGATGCCAATGCCTACTGGTTACCTAA |
| KanMX chk Reverse | 810 | CCCATATAAATCAGCATCCA |
| TOM70 pFA6 F5 | 1077 | TCAAGAAACTTTAGCTAAATTACGCGAACAGGGTTTAATGCGGATCCCCGGGTTAATTAA |
| TOM70 pFA6 R3 | 1078 | TTTGTCTTCTCCTAAAAGTTTTTAAGTTTATGTTTACTGTGAATTCGAGCTCGTTTAAAC |
| ILV2 NES Insertion | 4410 | ACAGACTGAATTACGTCATAAGCGTACAGGCGGTAAGCACTTAGCTTTAAAATTAGCTGGTTTAGATATTTAAGGCGCGCCACTTCTAAATAAGC |
| IDH1 pKT F5 | 1815 | TGACTTCACGAATGAAATCATCAACAAATTATCTACCATGGGTGACGGTGCTGGTTTA |
| IDH1 pKT R3 | 1816 | CACTTAAGTTGCAGAACAAAAAAAAGGGGAATTGTTTTCATCGATGAATTCGAGCTCG |
| **KO Primers** | | |
| ILV2 KO FW | 3380 | TAAGAGGAGATAAATACAACAGAATCAATTTTCAAGCAGATTGTACTGAGAGTGCACC |
| ILV2 KO REV | 3381 | ACTGAAAATGCTTTTGAAATAAATGTTTTTGAAATCTGTGCGGTATTTCACACCG |
| ILV2 KO chk D5 | 3382 | GTCTGTCAGTCGGCAC |
|  |  |  |
| ILV2 KO chk D3 | 3383 | GTTAAATTCGTATTGGCCACTG |
| DOA10 KO chk D3 | 2308 | GTGGCATTTAGTAGTCCAACTAGG |
| DOA10 KO chk D5 | 2061 | TCAACAATGGAACCCCCAACAATTATCTCA |
| DOA10 KO Fw | 2059 | TACCACTAATTGAATCAAAGAGACTAGAAGTGTGAAAGTCAGATTGTACTGAGAGTGCAC |
| DOA10 KO Rv | 2060 | TATGCTAGCATTCATTTTAAATGTAAGGAAGAAAACGCCTCTGTGCGGTATTTCACACCG |
| SAN1 KO check D5 | 1917 | TTGTATACTAGGTATTGCACCGCAGTCAGA |
| SAN1 KO chk D3 | 2281 | CCAACACTTGGTTTTCATGAC |
| SAN1 KO FW | 1915 | GTTTTCTCTCATAGTCTTGTAACCTCAGCTTTTGTTCATTAGATTGTACTGAGAGTGCAC |
| SAN1 KO REV | 1916 | GACATATTTTCATATTAACATACTTCAGAAGCGGTATTGTCTGTGCGGTATTTCACACCG |
| UBR1 KO chk D3 | 2307 | GCGAAGGATATGAAAATCAACC |
| UBR1 KO chk D5 | 1914 | TAACTTGCAGATAGTGACCATAAGGCAACT |
| UBR1 KO Fw | 1926 | AATCTTTACAGGTCACACAAATTACATAGAACATTCCAATAGATTGTACTGAGAGTGCAC |
| UBR1 KO Rv | 1913 | ACAAATATGTCAACTATAAAACATAGTAGAGGGCTTGAATCTGTGCGGTATTTCACACCG |
| PDR5 KO chk D3 | 2365 | GTTCGCCATTCGGACAGATAATG |
| PDR5 KO chk D5 | 1814 | CGGAACTCTTCTACGCCGTGGTACGATATC |
| PDR5 KO D3 | 1813 | TCTTGGTAAGTTTCTTTTCTTAACCAAATTCAAAATTCTACTGTGCGGTATTTCACACCG |
| PDR5 KO D5 | 1812 | AAGTTTTCGTATCCGCTCGTTCGAAAGACTTTAGACAAAAAGATTGTACTGAGAGTGCAC |
| **Cloning Primers** | | |
| DLD2 AA 1-35 R | 3399 | CTTCACCTTTAGACATGTTAATTAAACCAGCACCGTCACCATAGTTAACTCTTCTATAG |
| DLD2 AA36-530 F | 3401 | CTTAGTTTCGACGGATTCTAGAACTAGTGGATCCCCCGGGATGTATTCGACCAAGATAC |
| DLD2 pRS413 Fw | 2633 | CTTAGTTTCGACGGATTCTAGAACTAGTGGATCCCCCGGGATGCTAAGAAACATTTTGG |
| GFP pRS413-GPD RV | 2179 | TAATTACATGACTCGAGGTCGACGGTATCGATAAGCTTGATTATTTGTACAATTCATCC |
| ILV2 AAs 56-687 FW | 2180 | CTTAGTTTCGACGGATTCTAGAACTAGTGGATCCCCCGGGATGGAGCCTGCTCCAAGTTTC |
| ILV2 MTS FW | 2188 | CTTAGTTTCGACGGATTCTAGAACTAGTGGATCCCCCGGGATGATCAGACAATCTACGCT |
| ILV2 MTS RV | 2196 | CTTCACCTTTAGACATGTTAATTAAACCAGCACCGTCACCTGGCCTTTTAGAGGCTGG |
| ILV2 pRS413-GPD RV | 3714 | TAATTACATGACTCGAGGTCGACGGTATCGATAAGCTTGATCAGTGCTTACCGCCTGTAC |
| ILV2 MTS-Untagged RV | 4411 | TAATTACATGACTCGAGGTCGACGGTATCGATAAGCTTGATCATGGCCTTTTAGAGGCTGG |
| LAT1 AAs 29-482 FW | 2182 | CTTAGTTTCGACGGATTCTAGAACTAGTGGATCCCCCGGGATGGCATCGTACCCAGAGCACAC |
| LAT1 MTS FW | 2190 | CTTAGTTTCGACGGATTCTAGAACTAGTGGATCCCCCGGGATGTCTGCCTTTGTCAGGG |
| LAT1 MTS RV | 2198 | CTTCACCTTTAGACATGTTAATTAAACCAGCACCGTCACCGTAGCATCTCAATTGCAGTC |
| pKT adaptor FW | 2204 | GGTGACGGTGCTGGTTTA |
| COX15 AAs 66-486 FW | 2184 | CTTAGTTTCGACGGATTCTAGAACTAGTGGATCCCCCGGGATGAAACCACATGTTGCTTCAG |
| COX15 MTS FW | 2192 | CTTAGTTTCGACGGATTCTAGAACTAGTGGATCCCCCGGGATGCTTTTCAGAAACATAGAAG |
| COX15 MTS RV | 2200 | CTTCACCTTTAGACATGTTAATTAAACCAGCACCGTCACCAAAAACAGGGGAGGAGAGAG |
| GPD Fw (for sequencing) | 2277 | AAGACGGTAGGTATTGATTG |
| CYC Rv (for sequencing) | 2278 | GCGTACACGCGTTTGTAC |
