# Supplementary material for: A nuclear-based quality control pathway for non-imported mitochondrial proteins
Source: eLife. 2021 Mar 18;10:e61230. doi: 10.7554/eLife.61230 (PMC7993989; doi:10.7554/eLife.61230)
Supplement: Supplementary file 4. [file elife-61230-supp4.docx]

| **Plasmid** | **Construction** |
| --- | --- |
| pRS413-GPD-ILV2-GFP | pRS413-GPD cut w/ EcoRI + PCR product (AHY4042 gDNA amplified w/ 2188/2179) |
| pRS413-GPD-DLD2-GFP | pRS413-GPD cut w/ EcoRI + PCR product (AHY4951 gDNA amplified w/ 2633/2179) |
| pRS413-GPD-COX15-GFP | pRS413-GPD cut w/ EcoRI + PCR product (AHY3742 gDNA amplified w/ 2192/2179) |
| pRS413-GPD-LAT1-GFP | pRS413-GPD cut w/ EcoRI + PCR product (AHY3746 gDNA amplified w/ 2190/2179) |
| pRS413-GPD-N∆55ILV2-GFP | pRS413-GPD cut w/ EcoRI + PCR product (AHY4042 gDNA amplified w/ 2180/2179) |
| pRS413-GPD-N∆35DLD2-GFP | pRS413-GPD cut w/ EcoRI + PCR product (pRS413-GPD-DLD2-GFP amplified w/ 3401/2179) |
| pRS413-GPD-N∆65COX15-GFP | pRS413-GPD cut w/ EcoRI + PCR product (AHY3742 gDNA amplified w/ 2184/2179) |
| pRS413-GPD-N∆28LAT1-GFP | pRS413-GPD cut w/ EcoRI + PCR product (AHY3746 gDNA amplified w/ 2182/2179) |
| pRS413-GPD-MTS_ILV2_-GFP | pRS413-GPD cut w/ EcoRI + PCR product 1 (BY4741 gDNA amplified w/ 2188/2196) + PCR product 2 (pKT128 amplified w/ 2204/2179) |
| pRS413-GPD-MTS_DLD2_-GFP | pRS413-GPD cut w/ EcoRI + PCR product 1 (pRS413-GPD-DLD2-GFP amplified w/ 2633/3399) + PCR product 2 (pKT128 amplified w/ 2204/2179) |
| pRS413-GPD-MTS_COX15_-GFP | pRS413-GPD cut w/ EcoRI + PCR product 1 (BY4741 gDNA amplified w/ 2192/2200) + PCR product 2 (pKT128 amplified w/ 2204/2179) |
| pRS413-GPD-MTS_LAT1_-GFP | pRS413-GPD cut w/ EcoRI + PCR product 1 (BY4741 gDNA amplified w/ 2190/2198) + PCR product 2 (pKT128 amplified w/ 2204/2179) |
| pRS413-GPD-ILV2 | pRS413-GPD cut w/ EcoRI + PCR product (AHY4741 gDNA amplified w/ 2188/3714) |
| pRS413-GPD-N∆55ILV2 | pRS413-GPD cut w/ EcoRI + PCR product (AHY4741 gDNA amplified w/ 2180/3714) |
| pRS413-GPD-MTS_ILV2_ | pRS413-GPD cut w/ EcoRI + PCR product (AHY4741 gDNA amplified w/ 2188/4411) |
